# Supplementary material for: Comparative multiomics analysis of cell physiological state after culture in a basket bioreactor
Source: Sci Rep. 2022 Nov 23;12:20161. doi: 10.1038/s41598-022-24687-4 (PMC9686226; doi:10.1038/s41598-022-24687-4)
Supplement: Supplementary file 1 — Supplementary Information 1. [file 41598_2022_24687_MOESM1_ESM.zip › raw data/Proteomic raw data/6.KEGGenrichment/EG.vs.CG/ALL/pathway/src/03430.html]

map03430


- K04802

- Up regulated proteins

A0A0D9RLR8(Description:Proliferating cell nuclear antigen;Gene:PCNA;log2FC:1.0975)

- K10754, K10755, K10756

- Up regulated proteins

A0A0D9RWI0(Description:Replication factor C subunit 1;Gene:RFC1;log2FC:1.6366)

Close
